# Supplementary material for: Hydrostatic Filtration Enables Large-Scale Production of Outer Membrane Vesicles That Effectively Protect Chickens against Gallibacterium anatis
Source: Vaccines (Basel). 2020 Jan 23;8(1):40. doi: 10.3390/vaccines8010040 (PMC7158690; doi:10.3390/vaccines8010040)
Supplement: Supplementary file 1 [file vaccines-08-00040-s001.pdf]

| **Group:** **Animal nr:**<br>**Trial:** **Date:** | | **Score** | | | | |
|---|---|---|---|---|---|---|
| | | 0 | 1 | 2 | 3 | 4 |
| **Laying** | | | | | | |
| **Peritoneum** | Inflammatory reaction | | | | | |
| | Amount of exudate | | | | | |
| | Type of exudate | | | | | |
| | Transparency of peritoneum | | | | | |
| **Ovary** | Vascularisation | | | | | |
| | Pus | | | | | |
| | Degenerative/Regressive | | | | | |
| **Salpinx** | Circulation | | | | | |
| | Regression | | | | | |
| | Pus | | | | | |
| **Spleen** | Proliferation | | | | | |
| | Enlarged<br>Size in cm: lenght:_____ width______ | | | | | |
| **Other** | Liver | | | | | |
| | Lungs | | | | | |
| | | | | | | |

| | | |
|---|---|---|
| **P1** | Inflammatory reaction | 0: None<br>1: Local at the entrance<br>2: Around the ovary<br>3: In the omentum<br>4: All peritoneum |
| **P2** | Amount of exudate | 0: None<br>1: Sparse<br>2: Some<br>3: Abundant |
| **P3** | Transparency of peritoneum | 0: Clear<br>1: Unclear<br>2: Cloudy<br>3: Milky<br>4: Opaque |
| **P4** | Type of exudate | 0: None<br>1: Aqueous<br>2: Lump of pus<br>3: Flakes<br>4: Confluent |
| **O1** | Vascularisation | 0: None<br>1: Little<br>2: Moderate<br>3: Abundant |
| **O2** | Pus | 0: None<br>1: Local<br>2: Multifocal<br>3: Diffused |
| **O3** | Deformed/regressive follicle | 0: None<br>1: Few (1-2)<br>2: Less than half (3-4)<br>3: Over 4 |
| **S1** | Vascularisation | 0: None<br>1: Little<br>2: Moderate<br>3: Abundant |
| **S2** | Pus | 0: None<br>1: Local<br>2: Multifocal<br>3: Diffused |
| **S3** | Regression | 0: None/normal<br>1: Below average<br>2: More than average<br>3: Non-functional |
| **M1** | Lymphoid reaction | 0: None<br>1: Little proliferation<br>2: Distinct proliferation |
| **M2** | Enlargement | 0: None<br>1: Less than double size<br>2: More than double size<br>3: Atrophic/depleted |
